# High-Resolution Imaging of Tumor Spheroids and Organoids Enabled by Expansion Microscopy

**DOI:** 10.3389/fmolb.2020.00208

**Published:** 2020-09-24

**Authors:** Steven J. Edwards, Valentina Carannante, Kyra Kuhnigk, Henrik Ring, Tatsiana Tararuk, Finn Hallböök, Hans Blom, Björn Önfelt, Hjalmar Brismar

**Affiliations:** ^1^Science for Life Laboratory, Department of Applied Physics, KTH Royal Institute of Technology, Stockholm, Sweden; ^2^Department of Microbiology, Tumor and Cell Biology, Science for Life Laboratory, Karolinska Institutet, Stockholm, Sweden; ^3^Center for Hematology and Regenerative Medicine, Department of Medicine Huddinge, Karolinska Institutet, Stockholm, Sweden; ^4^Department of Neuroscience, BMC, Uppsala University, Uppsala, Sweden

**Keywords:** expansion, microscopy, spheroid, organoid, lightsheet, imaging

## Abstract

Three-dimensional cell cultures are able to better mimic the physiology and cellular environments found in tissues *in vivo* compared to cells grown in two dimensions. In order to study the structure and function of cells in 3-D cultures, light microscopy is frequently used. The preparation of 3-D cell cultures for light microscopy is often destructive, including physical sectioning of the samples, which can result in the loss of 3-D information. In order to probe the structure of 3-D cell cultures at high resolution, we have explored the use of expansion microscopy and compared it to a simple immersion clearing protocol. We provide a practical method for the study of spheroids, organoids and tumor-infiltrating immune cells at high resolution without the loss of spatial organization. Expanded samples are highly transparent, enabling high-resolution imaging over extended volumes by significantly reducing light scatter and absorption. In addition, the hydrogel-like nature of expanded samples enables homogenous antibody labeling of dense epitopes throughout the sample volume. The improved labeling and image quality achieved in expanded samples revealed details in the center of the organoid which were previously only observable following serial sectioning. In comparison to chemically cleared spheroids, the improved signal-to-background ratio of expanded samples greatly improved subsequent methods for image segmentation and analysis.

## Introduction

Multicellular tumor spheroids were first described in 1971 and were produced by culturing tumor cells in a non-adherent environment, resulting in the production of scaffold free, self-assembled, three dimensional cellular aggregates (Sutherland et al., 1971). The predominant advantage of 3-D cell cultures over cell monolayers concerns the ability of 3-D cultures to better mimic the *in vivo* cellular environment when compared to flat cells attached to hard plastic or glass surfaces (Pampaloni et al., 2007). Tumor spheroids are therefore being used to better predict the efficacy of anti-tumor treatments in high-throughput screening assays (Kunz-Schughart et al., 2004).

More recently, the development of tissue organoids has been embraced by the scientific community. In contrast to tumor spheroids, tissue organoids are produced from stem cells or organ progenitor cells which have been differentiated into multiple cell types with spatial organization and cellular functions mimicking those of the organ being modeled (Lancaster and Knoblich, 2014). Whilst sharing some of the advantages of tumor spheroids, the possibility of organoid production from human stem cells permits translational research and the study of developmental biology whilst reducing the ethical concerns and practical limitations associated with the study of explant tissue (Huch et al., 2017; Munsie et al., 2017).

Microscopic imaging of tissue organoids and spheroids is challenging due to the light scattering nature of their 3-D architecture. Live-imaging is further complicated by the slow nature of organoid development and the requirement for compatible cell culture systems at the microscope. Fast volumetric imaging with reduced light exposure is also preferred in order to reduce phototoxicity and photobleaching. To this extent, light sheet fluorescence microscopy has been the method of choice to study organoid and spheroid development over longer time scales (Pampaloni et al., 2014; Serra et al., 2019). End-point fluorescent imaging of fixed organoids and spheroids is typically limited to the outermost cell layers, due to light scatter and the poor penetrance of labels into the samples. The recent development of tissue clearing techniques has enabled 3-D volumetric imaging by greatly reducing light scatter, while improving the penetrance of labels to some extent through harsh permeabilization steps (Unnersjö-Jess et al., 2016).

More recently, expansion microscopy, an approach in which the specimen is physically expanded has been shown to permit super-resolution imaging on a conventional diffraction limited microscope (Chen et al., 2015; Ku et al., 2016; Tillberg et al., 2016). In addition to the physical expansion, samples become optically transparent due to the homogenous scattering of light by water molecules surrounding the hydrogel bound proteins. The porous nature of the hydrogel-protein hybrids may aid the diffusion of antibodies throughout the denatured sample. In this study we have demonstrated the advantages of combining expansion microscopy and immunolabeling of tumor spheroids and organoids compared to simple chemical immersion clearing methods in terms of (i) labeling quality, (ii) image quality as well as (iii) accuracy of subsequent image analysis.

## Materials and Methods

### Cell Line Maintenance

All cell lines were cultured at 37°C in a humidified (95%), 5% CO_2_ atmosphere and passaged before confluency. A498 renal carcinoma and a primary GBM culture #18 (Hägerstrand et al., 2006) stably expressing tdTomato following lentiviral transduction with plasmid #32904 (Addgene) were used to produce tumor spheroids. A498 renal carcinoma cell lines were cultured in RPMI 1640 GlutaMAX^TM^ medium (Thermo Fisher Scientific) supplemented with 10% fetal bovine serum (Thermo Fisher Scientific), 1 × MEM Non-Essential Amino Acid Solution (Sigma Aldrich) and 1% penicillin/streptomycin. GBM#18 cell lines were cultured in Minimum Essential Medium (Gibco) containing 2 mM L-Glutamine (Gibco) and 1% penicillin/streptomycin. MDCKII (ECACC 00062107) cells were cultured in EMEM (M2279, Sigma Aldrich) supplemented with 5% fetal bovine serum (Thermo Fisher Scientific), 2 mM L-Glutamine (Gibco) and 1% penicillin/streptomycin. NK92 GFP malignant non-Hodgkin’s lymphoma cell line was maintained at 37°C in 5% CO_2_ in RPMI 1640 with L-glutamine (Thermo Fisher Scientific) supplemented with 10% fetal bovine serum (Sigma Aldrich), 1× MEM Non-Essential Amino Acid Solution (Sigma-Aldrich) and 10 mM Hepes (Sigma-Aldrich). IL-2 (R&D System) was added to NK92 GFP culture every 2 days at final concentration of 500 U/ml to induce cell proliferation (Sutlu et al., 2012).

### Cell Line Transduction

The stable A498 cell line transduced with RFP was obtained as described elsewhere (Barde et al., 2010). To summarize, HEK293T Lenti-X cells were co-transfected with a pLenti-CMV-MCS-RFP-SV-puro and the vectors pMD2g and pSPAX2, which encode the envelope and packaging proteins, respectively. Lentiviral particles were harvested and the titration was performed on the HEK293T-Lenti-X cells by flow cytometry. Finally, low passage A498 cells were transduced with a range of viral loads. The cells were tested to quantify the expression efficiency of RFP and sorted by flow cytometry. Cells were maintained in culture in 8 μg/mL puromycin.

### Cell Line Transfection

A498 renal carcinoma cells were transfected with CellLight^®^ Mitochondria-GFP, BacMam 2.0 (Thermo Fisher Scientific) overnight with 50 baculovirus particles per cell.

### Production of Tumor Spheroids

Agarose multi-well 3-D petri dishes were produced by adding 500 μL of a 2% Agarose dissolved in sterile saline solution (0.9% [w/v] NaCl) to a 3-D Petri Dishes^®^ 12–256 mould (Micro Tissues Inc.). 150 μL of A498 or GBM#18 cell suspension was seeded into 3-D petri dishes (Micro Tissues Inc.) at a concentration of 500,000 cells/mL in a 12-well plate. Cells were allowed to settle for 30 min and 2 mL of cell culture medium was added to the well. Cell spheroids formed over 4 days in a cell incubator.

### Production of MDCKII Cysts

MDCK II cysts were produced by adding a single cell suspension of MDCK II cells to a neutralized Type 1 collagen solution (PureCol EZ Gel, Advanced Biomaterials) at a concentration of approximately 5 × 10^4^ cells/mL. The collagen was allowed to polymerize as droplets on a 60 mm petri dish at 37°C for 60 min. Medium was added and replaced every third day. Cysts were fully formed and harvested after approximately 10 days. Prior to fixation, 1 mL of media was removed from the well and 5 mg of collagenase (5 mg/mL, C7926 Sigma) added. The media containing collagenase was returned to the well and the collagen was digested for 10 min at 37°C.

### Production of Retinal Organoids and Electroporation

hESC culture. The HS980 cell line was maintained on laminin (LN521-03, Biolamina, Sundbyberg, Sweden)-coated plates with NutriStem hESC XF (05-100-1A, Saveen & Werner, Limhamn, Sweden) (Rodin et al., 2014). Cells were passaged every 3–4 days at 90% confluence. The HS980 cell line was induced to differentiate toward neural retina following the protocol published by Zhong et al. (2014).

Early stages of retinal differentiation. On day 0 (d0) of differentiation, HS980 cells 90% confluency were detached by incubation with PBS at 37°C, for 5 min and then scraped using a 1 mL pipette tip. Small aggregates were transferred to Ultralow Attachment (ULA) plates and cultured in suspension with NutriStem (NS) and 10 μM Blebbistatin (B0560, Sigma-Aldrich) to induce aggregate formation. NS was gradually replaced with neural induction medium (NIM) containing DMEM/F12 (1:1), 1% N2 supplement (17502-001, Gibco, Thermo Fisher Scientific), 1× minimum essential media-non-essential amino acids, 2 mg/mL heparin (H3393, Sigma-Aldrich), with a 3:1 ratio of NS/NIM on d1, 1:1 on d2 and 100% NIM on d3. On d7, aggregates were seeded onto Matrigel (734–0269, growth-factor-reduced; VWR, Stockholm, Sweden)-coated dishes containing NIM and switched to DMEM/F12 (3:1) supplemented with 2% B27 (12587-010, without vitamin A, Gibco), 1× NEAA and 1% antibiotic–antimycotic (15240-062, Gibco) on d16. Thereafter, the medium was changed daily.

Formation of Retinal organoids. On days 25–28 of differentiation, horseshoe-shaped neural retina domains were manually detached under inverted microscope, collected and cultured in suspension at 37°C in a humidified 5% CO_2_ incubator in DMEM/F12 (3:1) supplemented with 2% B27, 1× NEAA, and 1% antibiotic–antimycotic, where they gradually formed 3D retinal organoids. Thereafter, the medium was changed twice a week. For long-term suspension culture, the medium was supplemented with 10% fetal bovine serum (16000-044, Gibco), 100 mM Taurine (T0625-100G, Sigma-Aldrich) and 2 mM GlutaMAX (35050-038, Gibco) beginning on d41.

Electroporation (EP). Retinoids were electroporated to introduce GFP-expressing and in order to achieve stable expression, a piggyBack-vector system was used, consisting of a GFP-reporter gene-construct with a piggyBack transposon cassette containing the ubiquitously active chicken β-actin (CAG) promoter with the cytomegalovirus early enhancer that drives GFP expression (pB CAG-GFP) (Blixt and Hallböök, 2016). The GFP vector was electroporated together with a helper transposase vector (CAG-pBase) encoding an integrase that catalyzes the integration of the GFP reporter cassette into the genome of electroporated cells, establishing cells with robust and stable GFP expression. The GFP was localized in the plasma membrane. Retinoids were electroporated on d30 of differentiation. Three to five retinoids were electroporated in an electroporation cuvette with a 1 mm gap using an ECM 830 SW electroporator (BTX, Holliston, MA, United States). The electroporator was set to 15V, 5 pulses of 50 ms and 1 s interval between pulses. DNA concentration of each plasmid was 1.6 μg/μL.

### Production of Tumor Spheroid and NK-92 Cell Co-cultures

Agarose hydrogels containing 256 micro-wells were obtained with 3-D Petri Dish technology (#12–256-Small, MicroTissues, Inc.) following the manufacturers instruction. A498 RFP renal cell carcinoma cells were seeded in the agarose 3-D Petri Dish (75000 cells/agarose gel) and maintained in complete medium at 37°C, 5% CO_2_ for 96 h to allow spheroid formation. NK92 GFP cells were added to pre-formed A498 RFP renal carcinoma spheroid at E:T ratio of 1:1 and maintained at 37°C, 5% CO_2_ for 2 h to allow NK92 cell-infiltration before fixation.

### Pre- expansion Staining and Chemical Immersion Clearing

GBM culture #18 spheroids were fixed in 4% PFA in PBS for 15 min before being washed in PBS and stored at 4°C. Spheroids were permeabilized for 15 min in 0.5% Triton X-100 in PBS and blocked for 1 h in PBS containing 5% BSA, 100 mM Glycine, 0.2% Triton X-100 and 0.05% Tween-20. Spheroids were stained overnight in anti-tdTomato polyclonal antibody (1:50, Sicgen) at 37°C in blocking solution and washed 3 times for 20 min in blocking solution. Spheroids were incubated overnight in secondary antibody donkey anti-sheep Star635p (1:25, Abberior) and DAPI (1:20,000) at 37°C and washed 3 times for 20 min in blocking solution. Spheroids were immersion cleared in Omnipaque 350 (iohexol 755 mg/mL, Refractive Index = 1.458, GE healthcare) by embedding the stained spheroids into an acrylamide gel containing 4% acrylamide (w/w), 0.05% (w/w) bis-acrylamide 0.2% (w/w) TEMED and 0.2% (w/w) APS in PBS. For chemical immersion clearing, the spheroids were incubated in Omnipaque 350 2 times for 2 h before proceeding with imaging.

### Anchoring and Embedding

GBM culture #18 and A498 renal carcinoma cell spheroids were fixed in 4% PFA, 0.05% Glutaraldehyde and 0.3% Triton X-100 in PBS for 15 min before being washed in PBS and stored at 4°C. MDCKII cysts, retinal organoids and tumor spheroid and NK-92 cell co-cultures were fixed in 4% PFA in PBS for 15 min and washed in PBS. Fixed samples were anchor treated overnight in Acryloyl X-SE (0.1 mg/mL, Thermo-Fisher) in PBS at room temperature. Samples were washed 2 times for 15 min in PBS and subsequently anchored in MA-NHS (25 mM in PBS, Sigma-Aldrich) for 1 h at room temperature. Samples were once again washed in PBS 2 times for 15 min. Samples were incubated in a monomer solution containing [1 × PBS, 2 M NaCl, 8.625% (w/w) sodium acrylate, 2.5% (w/w) acrylamide, 0.15% (w/w) N,N’-methylenebisacrylamide] for 30 min on ice and the samples were transferred to a polymerization chamber composed of a microscopy slide and cover slip separated by three No. 1.5 coverslips as spacers. For a more detailed description of the chamber see Asano et al. (2018).

The monomer solution in the polymerization chamber was replaced with fresh monomer solution containing TEMED (0.2% w/w), APS (0.2% w/w) and 4-hydroxy-2,2,6,6-tetramethylpiperidin-1-oxyl (4-hydroxy-TEMPO) (0.01% w/w). The monomer solution was allowed to polymerize for 2 h at 37°C in a humidified incubator.

### Denaturation, Staining and Expansion

For spheroids containing endogenous reporters (eGFP), the gels were removed from the polymerization chamber and equilibrated in a digestion buffer containing (50 mM Tris (pH 8), 1 mM EDTA, 0.5% Triton X-100, 1 M NaCl) for 15 min. The digestion buffer was replaced with fresh buffer containing proteinase-K (8 units/mL, New England Biolabs) for 3 h at room temperature. The digested sample was washed in PBS 3 times for 15 min before being expanded in deionized (DI) water for at least 45 min with three periodic changes to fresh DI water.

For heat disruption and subsequent staining, the gels were removed from the gelling chamber and equilibrated in a heat disruption buffer containing 100 mM Tris base, 5% (w/v) Triton X−100, 1% (w/v) SDS, in water. Gels were heated to 70°C for 1 h followed by 95°C for 1 h before being washed 3 times for 15 min in PBS. GBM culture #18 spheroids were stained with anti-tdTomato (Sicgen) primary antibodies overnight at 37°C in blocking solution, washed 3 times for 20 min in blocking solution and stained with donkey anti-sheep Star635p (Abberior^®^) secondary antibodies overnight at 37°C in blocking solution. A498 spheroids were stained with the following antibodies: Anti-Mic60 (1:100, proteintech^®^), Anti-P-histone (1:100, proteintech^®^), Anti-TUBA4A (1:100, Sigma-Aldrich), Anti-ARFGAP1 (1:100, ATLAS ANTIBODIES) in PBST overnight at 37°C, washed 3 times for 20 min in PBST and stained with Anti-rabbit Star635p (1:100, Abberior^®^) or Anti-goat Star635p (1:100, Abberior^®^) secondary antibodies in PBST overnight at 37°C. Spheroids were co-stained with DAPI (1:1000) in the secondary antibody solution.

Wheat-germ Agglutinin (WGA) was conjugated to Alexa Fluor 647, diluted (1:50) in PBST and stained overnight at 37°C. For a full list of staining conditions see Supplementary Table 1.

Samples were washed with 0.1% Triton X-100 in PBS (PBST) 3 times for 15 min after secondary antibody staining and expanded in DI water for at least 45 min with three periodic changes to fresh DI water.

### Light Sheet Microscopy

Expanded and cleared samples were glued (Super Glue Precision, Loctite) to a metal rod and introduced to the chamber of a Zeiss Light sheet Z1 microscope containing DI water (expanded sample) or Omnipaque 350 with a refractive index of 1.458 (GE Healthcare) (cleared sample). Fluorescence was excited from one side using a 10 × 0.2 NA illumination objective and detected using a 10 × 0.4 NA water dipping objective, 20 × 1.0 NA water dipping objective with the collar set to a refractive index of 1.33 (expanded sample) or 20 × 1.0 NA clearing dipping objective with the collar set to a refractive index of 1.458 (cleared sample).

### Image Processing and Analysis

For semi-automated nuclei quantification in cleared and expanded spheroids, the “Surface Object Creation” tool of Imaris 9.1 (Bitplane) was used. Surfaces were created with a manual threshold which included all voxels above background. Close nuclei were separated using a watershed-based surface splitting algorithm and a seed point diameter of 8 μm (cleared) and 25 μm (expanded). Significance *p* < 0.001 represented as ^∗∗∗^. The signal-to-background ratio was defined as the ratio between the average signal intensity in a region of interest drawn in the cell nuclei and the cell cytosol.

## Results

**FIGURE 1:**
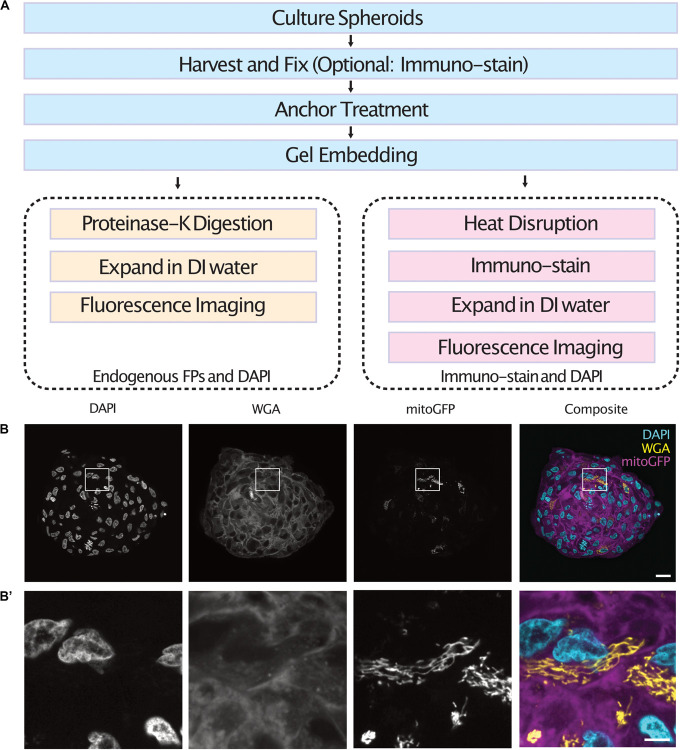
Workflow of the expansion microscopy protocols demonstrated here for different labeling strategies **(A)**. Central section of an expanded tumor spheroid transfected with a mitochondria-tagged GFP and stained with DAPI and WGA **(B)** and inserts **(B’)**. Scale bar: 100 μm **(B)** and 20 μm **(B’)**. FPs, Fluorescent Proteins.

Cell spheroids and organoids were expanded following one of two protocols depending on the labeling method used (Figure 1A). In both cases, spheroids were fixed, anchored and gel embedded to produce a protein-hydrogel hybrid. For spheroids containing cells expressing endogenous fluorescent proteins, digestion of the sample was performed using proteinase-K as the endogenous fluorescence was preserved after denaturation (Tillberg et al., 2016). For immuno-staining of spheroids, the samples were heat disrupted at 70°C for 1 h followed by 95°C for 1 h. Following enzymatic digestion or heat disruption, the samples were allowed to expand in DI water before fluorescence imaging in a light sheet fluorescence microscope. We demonstrate the use of this protocol by expanding and imaging tumor spheroids containing a subset of cells expressing a mitochondria-targeted GFP which were co-stained with DAPI and WGA (Figures 1B,B’ and Supplementary Videos 1, 2).

**FIGURE 2:**
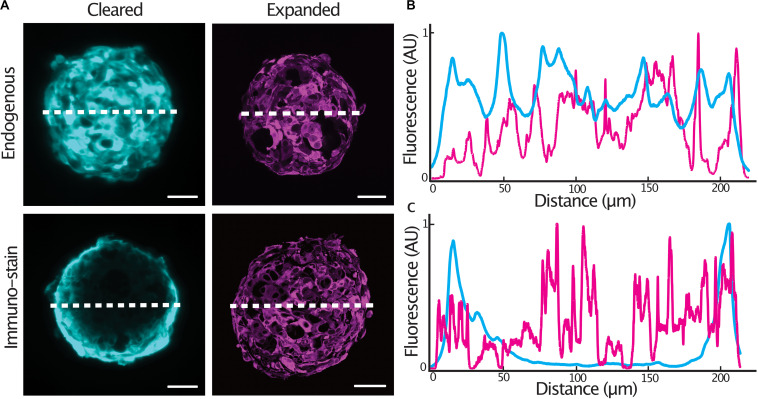
Expansion microscopy improves antibody penetration into tumor spheroids. Central sections of expanded GBM#18 tumor spheroids expressing a cytosolic tdTomato fluorescent protein **(A)**, line profile of fluorescent intensity through the center of a cleared or expanded spheroid (endogenous tdTomato signal) **(B)**, line profile of fluorescent intensity through the center of a cleared or expanded spheroid (anti-tdTomato immuno-stain signal) **(C)**. Fluorescence (AU) was normalized and distance of the expanded spheroids was normalized to that of the cleared spheroid. Scale bar: 50 μm (cleared), 200 μm (expanded).

In order to achieve homogenous immunolabeling of 3-D tumor spheroids, we hypothesized that immunolabeling post heat-disruption would improve antibody penetration into the samples. We compared staining quality in glioblastoma cell spheroids expressing a cytosolic tdTomato fluorescent protein as antibody penetration can be problematic when an epitope is abundantly expressed (Murray et al., 2015). Spheroids were fixed and either, stained with an anti-tdTomato primary antibody and cleared, or, stained following heat-disruption and expanded. Endogenous tdTomato fluorescence was homogenous throughout an optical section at the center of both cleared and expanded spheroids (Figures 2A,B). In spheroids stained after fixation, antibody penetration was confined to the outer cell layers of the spheroid. Immunolabeling was much improved in expanded spheroids that were stained following heat-disruption (Figures 2A,C), possibly due to the more porous nature of the expanded protein-hydrogel.

**FIGURE 3:**
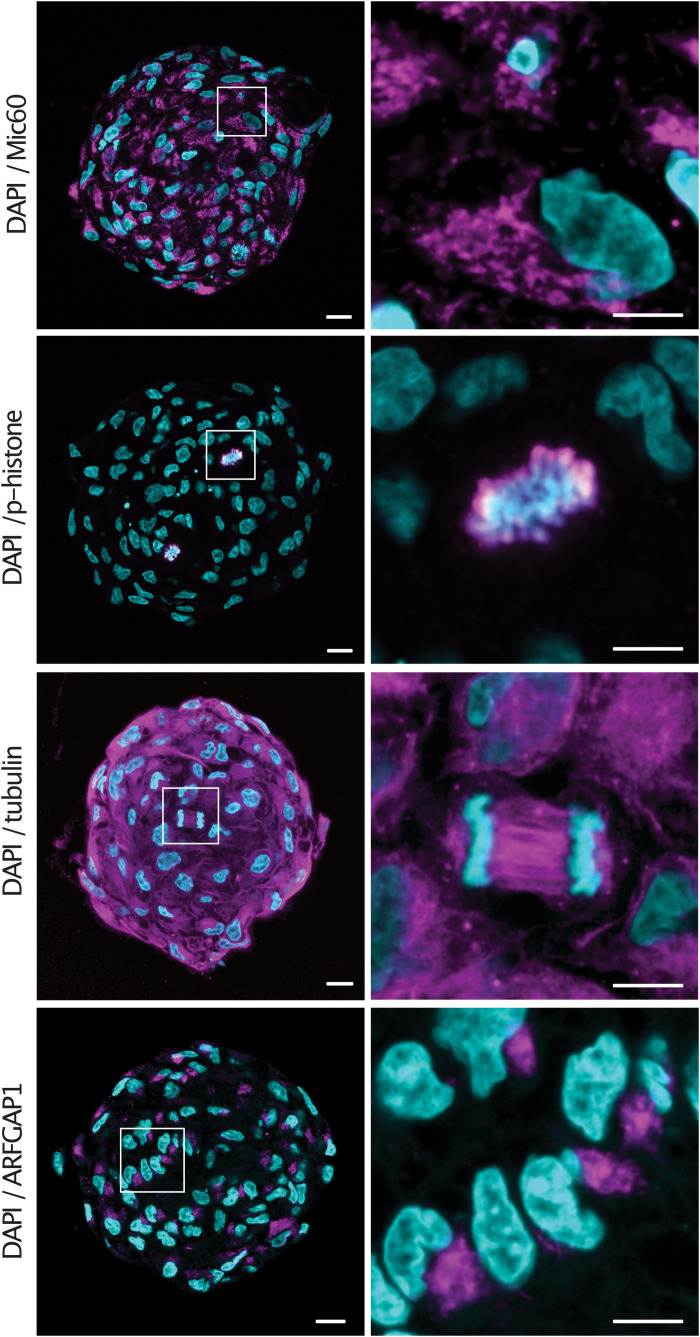
Epitopes are preserved in expanded spheroids following denaturation at 95°C. Examples of central sections of tumor spheroids stained with anti-Mic60, anti-p-histone, anti-tubulin and anti-ARFGAP1 primary antibodies (magenta) and counterstained with DAPI (cyan). Scale bars: 50 μm and 20 μm (insert).

Having observed the improved immunolabeling in expanded samples, we sought to validate the protocol for a number of commonly used antibodies. Spheroids were embedded and denatured at 95°C for 1 h before being stained with a diverse range of primary antibodies with targets in the cytosol as well as multiple organelles. Spheroids were stained with Mic60 (mitochondrial inner membrane), phospho-histone (chromatin), tubulin (cytoskeleton) and ARFGAP1 (golgi) primary antibodies and counterstained with DAPI. All antibodies showed expected staining patterns as well as homogenous staining throughout the sample (Figure 3). Notably, condensed chromosomes and individual spindle fibers could be resolved in dividing cells throughout the expanded spheroids.

**FIGURE 4:**
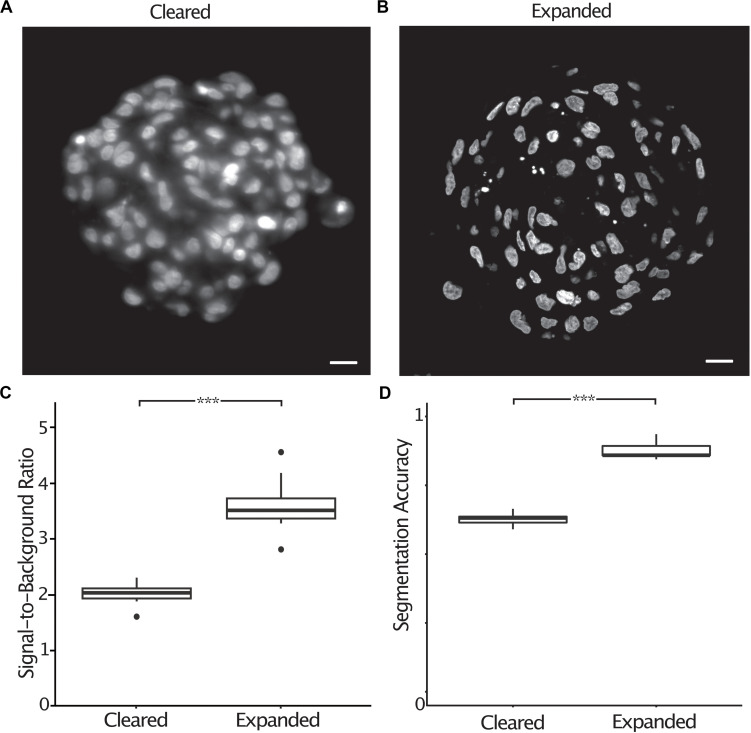
The signal-to-background ratio is improved in expanded compared to cleared spheroids, increasing the accuracy of semi-automatic nuclei segmentation. Central section of a cleared A498 spheroid stained with DAPI **(A)**, central section of an expanded A498 spheroid stained with DAPI **(B)**, signal-to-background ratio measured in cleared and expanded spheroids (*p* < 0.001, represented as ***, unpaired *t*-test) **(C)**, segmentation accuracy of DAPI stained nuclei in cleared and expanded spheroids (*p* < 0.001, represented as ***, unpaired *t*-test) **(D)**. Scale bar: 20 μm (cleared) and 50 μm (expanded).

Most image processing and analysis methods such as fluorescence colocalization and segmentation are easier to perform when images have a high signal-to-background ratio. Expanded spheroids stained with DAPI showed an improved signal-to-background ratio by a factor of 1.8 (2.0 ± 0.07 s.e.m. to 3.6 ± 0.17 s.e.m.) compared to cleared specimens (Figures 4A–C). To demonstrate the improved ability to segment expanded samples due to the improved signal-to-background ratio and improved resolution, we semi-automatically counted nuclei in chemically cleared and expanded spheroids and compared the number of nuclei to a “ground truth” measurement performed by an expert annotator. We defined the segmentation accuracy as the number of semi-automatically counted nuclei divided by the “ground truth” count. In cleared spheroids, semi-automatic counting gave a segmentation accuracy of 0.63 (± 0.010 s.e.m.) whereas in expanded spheroids the accuracy increased to 0.88 (± 0.013 s.e.m.) (Figure 4D). It was clear that image segmentation approaches using a watershed algorithm to split nuclei in close proximity were more successful in expanded compared to chemically cleared samples.

**FIGURE 5:**
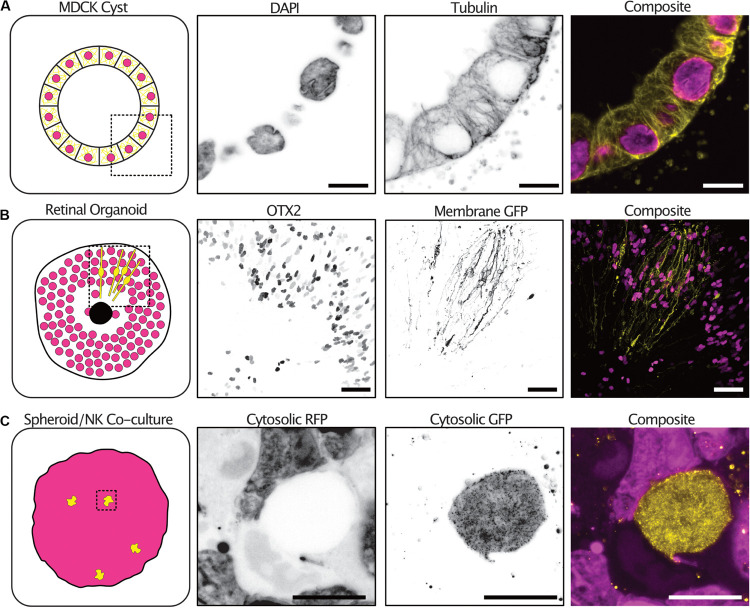
The expansion microscopy protocol can be applied to a diverse range of 3-D cultures. Examples of expansion microscopy applied to an MDCK II cyst stained with DAPI (magenta) and an anti-tubulin antibody (yellow) – single plane **(A)** (Scale bar 30 μm), retinal organoid stained with an OTX2 antibody (magenta) and transfected with a membrane targeted GFP (yellow) – max intensity projection **(B)** (Scale bar 200 μm), an A498 cell spheroid expressing cytosolic RFP (magenta) and NK-92 expressing cytosolic GFP (yellow) co-culture – max intensity projection **(C)** (Scale bar 50 μm).

Tumor spheroid mono-cultures encompass only a small fraction of the currently used 3-D culture models. We therefore sought to demonstrate the expansion protocol on spheroid co-cultures and organoids with different sizes, extracellular compositions and cellular subtypes. Kidney epithelial cells (MDCKII) grown in an extracellular matrix-like environment differentiate into a spherical formation containing polarized cells termed cysts. The cysts reproduce many of the features of an epithelial tissue *in vivo*. 3-D imaging of cysts is typically limited by the light scattering nature of the collagen matrix surrounding the cysts. Using expansion, we were able to image cysts at all depths and with subcellular resolution sufficient to resolve individual tubulin filaments (Figure 5A and Supplementary Figure 1 and Supplementary Video 3).

Furthermore, retinal organoids derived from human embryonic stem cells mimic human retinogenesis. They reproduce the formation of organized layered structures and transcription factor markers of typical retinal cell types. We grew, antibody stained and expanded retinoids that were electroporated with a membrane targeted GFP to observe the distribution of cells types and cellular morphologies. The transcription factor OTX2 which is expressed in immature photoreceptors and in mature bipolar cells was distributed at the periphery of the retinoid and differential expression could be seen between neighboring cells (Glubrecht et al., 2009). In addition, the electroporation with a membrane targeted GFP labeled cells with a highly polarized morphology sending projections both to the center of the organoid and the periphery (Figure 5B and Supplementary Video 4).

Natural Killer (NK) cells are innate lymphoid cells that rapidly perform cell-mediated cytotoxicity and release pro-inflammatory cytokines in response to tumor transformed cells (Cooper et al., 2001). Tumor spheroids partially mimic the tumor microenvironment *in vitro*, providing a better system for studying NK infiltration and behavior in solid tumors (Costa et al., 2016). We grew tumor spheroids and added NK92 cells expressing a cytosolic GFP to the culture. NK92 cells were allowed to infiltrate the spheroids for 2 h before the co-cultures were fixed and expanded. To increase the GFP signal, anti-GFP antibodies were used after digestion with proteinase-K. NK92 cells could be seen throughout the spheroid volume and their morphology visualized (Figure 5C and Supplementary Video 5).

## Discussion

As tissue culture methods advance into the third dimension, sample preparation methods as well as microscopy techniques must be adapted and improved. This work builds upon tissue clearing approaches which mitigate light scatter and enables high-resolution imaging of 3-D samples without the need of physical sectioning. Previous attempts to chemically clear and image tumor spheroids have been limited, particularly when homogenous staining with large labels such as antibodies is required. Improved antibody penetration has also been observed with clearing methods such as CLARITY, CUBIC and iDISCO but homogenous labeling of thick samples is still problematic, especially when the epitope is abundantly expressed (Chung et al., 2013; Renier et al., 2014; Susaki et al., 2015). The expansion microscopy protocol evaluated here enabled homogenous staining of tumor spheroids and permitted high-resolution imaging of large three-dimensional organoids with a high signal-to-background ratio using optical sectioning microscopy.

In addition to the improved signal-to-background ratio, expansion microscopy improves the effective resolution that can be achieved with any given objective. The resolution improvement is achieved equally in both the lateral and axial direction as the volumetric expansion of samples has been shown to be homogenous even at the nanoscale (Pesce et al., 2019). As the resolution of a given microscope is typically far worse in the axial direction, cell structures are more difficult to segment if they are in close proximity and oriented axial to the detection objective. Expansion microscopy is therefore particularly beneficial when the segmentation accuracy is limited by the axial resolution of the microscope. It is likely that additional methods to improve the axial resolution of an image, such as multi-view imaging and subsequent deconvolution or deep learning methods such as content-aware image restoration (CARE) could improve the segmentation accuracy further (Preibisch et al., 2014; Weigert et al., 2018).

Current expansion protocols are low-throughput. In our hands, it is possible to perform the protocol in 2 days whilst imaging tens of spheroids using light sheet fluorescence microscopy. The imaging time would increase dramatically if a point-scanning based microscopy method such as confocal microscopy was used. Further developments would allow for the expansion of spheroids and organoids grown in individual wells of glass-bottomed multi-well plates to be expanded and imaged in the well. This would also allow the spheroids or organoids to be screened in a high-throughput manner with small molecule compounds and imaged without the requirement for transfers and associated losses. Open top light sheet microscopes designed for fast 3-D imaging of cleared biopsies could be adapted for expanded samples and provide fast volumetric imaging (Glaser et al., 2019).

The expansion microscopy protocols used here have been shown to provide isotropic expansion on protein structures down to 10 nm (Pesce et al., 2019). Although most antibodies worked in our hands, some epitopes which are heat-sensitive may be denatured during the protocol. For unknown epitopes for which the staining pattern is completely unknown, we recommend that staining is performed on sectioned tissue and compared with expanded samples.

Spheroids are praised for their ability to more accurately reproduce the gradients of micronutrients such as metabolites, catabolites and oxygenation found in the tumor microenvironment (Friedrich et al., 2007). Flow cytometry is the standard method to quantify protein expression at a single cell level, and it has been recently used to study tumor spheroids (Olofsson et al., 2018). However, the technique requires dissociation of the spheroid into a single cell suspension which results in the loss of spatial information such as the cells microenvironment. We envision a workflow in which single cell analysis could be performed on spheroids and organoids through single-cell segmentation, improved substantially by the high signal-to-background ratio achieved when imaging expanded samples (Mosaliganti et al., 2012).

## Data Availability Statement

The raw data supporting the conclusions of this article will be made available by the authors, without undue reservation.

## Author Contributions

SE and HB conceived and planned the experiments. SE, VC, KK, HR, and TT carried out the experiments and contributed to sample preparation. SE took the lead in writing the manuscript. All authors contributed to the interpretation of the results and provided critical feedback and helped shape the research, analysis and manuscript.

## Conflict of Interest

The authors declare that the research was conducted in the absence of any commercial or financial relationships that could be construed as a potential conflict of interest.

## References

[B1] Asano S. M. Gao R. Wassie A. T. Tillberg P. W. Chen F. Boyden E. S. (2018). Expansion microscopy: protocols for imaging proteins and RNA in cells and tissues. *Curr. Protoc. Cell Biol.* 80:e56. 10.1002/cpcb.56 PMC6158110 30070431

[B2] Barde I. Salmon P. Trono D. (2010). Production and titration of lentiviral vectors. *Curr. Protoc. Neurosci.* Chater4:Unit12.10. 10.1002/0471142301.ns0421s53 20938923

[B3] Blixt M. K. E. Hallböök F. (2016). A regulatory sequence from the retinoid X receptor γ gene directs expression to horizontal cells and photoreceptors in the embryonic chicken retina. *Mol. Vis.* 22 1405– 1420. 28003731 PMC5166796

[B4] Chen F. Tillberg P. W. Boyden E. S. (2015). Expansion microscopy. *Science* 347 543–548. 25592419 10.1126/science.1260088 PMC4312537

[B5] Chung K. Wallace J. Kim S.-Y. Kalyanasundaram S. Andalman A. S. Davidson T. J. (2013). Structural and molecular interrogation of intact biological systems. *Nature* 497 332–337. 23575631 10.1038/nature12107 PMC4092167

[B6] Cooper M. A. Fehniger T. A. Caligiuri M. A. (2001). The biology of human natural killer-cell subsets. *Trends Immunol.* 22 633–640. 10.1016/s1471-4906(01)02060-9 11698225

[B7] Costa E. C. Moreira A. F. de Melo-Diogo D. Gaspar V. M. Carvalho M. P. Correia I. J. (2016). 3D tumor spheroids: an overview on the tools and techniques used for their analysis. *Biotechnol. Adv.* 34 1427–1441. 10.1016/j.biotechadv.2016.11.002 27845258

[B8] Friedrich J. Ebner R. Kunz-Schughart P. D. L. A. (2007). Experimental anti-tumor therapy in 3-D: spheroids – old hat or new challenge? *Int. J. Radiat. Biol.* 83 849–871. 10.1080/09553000701727531 18058370

[B9] Glaser A. K. Reder N. P. Chen Y. Yin C. Wei L. Kang S. (2019). Multi-immersion open-top light-sheet microscope for high-throughput imaging of cleared tissues. *Nat. Commun.* 10 1–8. 31273194 10.1038/s41467-019-10534-0 PMC6609674

[B10] Glubrecht D. D. Kim J.-H. Russell L. Bamforth J. S. Godbout R. (2009). Differential CRX and OTX2 expression in human retina and retinoblastoma. *J. Neurochem.* 111 250–263. 10.1111/j.1471-4159.2009.06322.x 19686387 PMC3726384

[B11] Hägerstrand D. Hesselager G. Achterberg S. Wickenberg Bolin U. Kowanetz M. Kastemar M. (2006). Characterization of an imatinib-sensitive subset of high-grade human glioma cultures. *Oncogene* 25 4913–4922. 10.1038/sj.onc.1209497 16547494

[B12] Huch M. Knoblich J. A. Lutolf M. P. Martinez-Arias A. (2017). The hope and the hype of organoid research. *Development* 144 938–941. 10.1242/dev.150201 28292837

[B13] Ku T. Swaney J. Park J.-Y. Albanese A. Murray E. Cho J. H. (2016). Multiplexed and scalable super-resolution imaging of three-dimensional protein localization in size-adjustable tissues. *Nat. Biotechnol.* 34 973–981. 10.1038/nbt.3641 27454740 PMC5070610

[B14] Kunz-Schughart L. A. Freyer J. P. Hofstaedter F. Ebner R. (2004). The use of 3-D cultures for high-throughput screening: the multicellular spheroid model. *J. Biomol. Screen.* 9 273–285. 10.1177/1087057104265040 15191644

[B15] Lancaster M. A. Knoblich J. A. (2014). Organogenesis in a dish: modeling development and disease using organoid technologies. *Science* 345:1247125. 10.1126/science.1247125 25035496

[B16] Mosaliganti K. R. Noche R. R. Xiong F. Swinburne I. A. Megason S. G. (2012). ACME: automated cell morphology extractor for comprehensive reconstruction of cell membranes. *PLoS Comput. Biol.* 8:e1002780. 10.1371/journal.pcbi.1002780 23236265 PMC3516542

[B17] Munsie M. Hyun I. Sugarman J. (2017). Ethical issues in human organoid and gastruloid research. *Development* 144 942–945. 10.1242/dev.140111 28292838

[B18] Murray E. Cho J. H. Goodwin D. Ku T. Swaney J. Kim S.-Y. (2015). Simple, scalable proteomic imaging for high-dimensional profiling of intact systems. *Cell* 163 1500–1514. 10.1016/j.cell.2015.11.025 26638076 PMC5275966

[B19] Olofsson K. Carannante V. Ohlin M. Frisk T. Kushiro K. Takai M. (2018). Acoustic formation of multicellular tumor spheroids enabling on-chip functional and structural imaging. *Lab. Chip.* 18 2466–2476. 10.1039/c8lc00537k 30033460

[B20] Pampaloni F. Berge U. Marmaras A. Horvath P. Kroschewski R. Stelzer E. H. K. (2014). Tissue-culture light sheet fluorescence microscopy (TC-LSFM) allows long-term imaging of three-dimensional cell cultures under controlled conditions. *Integr. Biol. Quant. Biosci. Nano Macro.* 6 988–998. 10.1039/c4ib00121d 25183478

[B21] Pampaloni F. Reynaud E. G. Stelzer E. H. K. (2007). The third dimension bridges the gap between cell culture and live tissue. *Nat. Rev. Mol. Cell Biol.* 8 839–845. 10.1038/nrm2236 17684528

[B22] Pesce L. Cozzolino M. Lanzanò L. Diaspro A. Bianchini P. (2019). Measuring expansion from macro- to nanoscale using NPC as intrinsic reporter. *J. Biophotonics.* 12:e201900018. 10.1002/jbio.201900018 PMC7065622 30980601

[B23] Preibisch S. Amat F. Stamataki E. Sarov M. Singer R. H. Myers E. (2014). Efficient Bayesian-based multiview deconvolution. *Nat. Methods* 11 645–648. 10.1038/nmeth.2929 24747812 PMC4153441

[B24] Renier N. Wu Z. Simon D. J. Yang J. Ariel P. Tessier-Lavigne M. (2014). iDISCO: a simple, rapid method to immunolabel large tissue samples for volume imaging. *Cell* 159 896–910. 10.1016/j.cell.2014.10.010 25417164

[B25] Rodin S. Antonsson L. Hovatta O. Tryggvason K. (2014). Monolayer culturing and cloning of human pluripotent stem cells on laminin-521–based matrices under xeno-free and chemically defined conditions. *Nat. Protoc.* 9 2354–2368. 10.1038/nprot.2014.159 25211513

[B26] Serra D. Mayr U. Boni A. Lukonin I. Rempfler M. Challet Meylan L. (2019). Self-organization and symmetry breaking in intestinal organoid development. *Nature* 569 66–72. 10.1038/s41586-019-1146-y 31019299 PMC6544541

[B27] Susaki E. A. Tainaka K. Perrin D. Yukinaga H. Kuno A. Ueda H. R. (2015). Advanced CUBIC protocols for whole-brain and whole-body clearing and imaging. *Nat. Protoc.* 10 1709–1727. 10.1038/nprot.2015.085 26448360

[B28] Sutherland R. M. McCredie J. A. Inch W. R. (1971). Growth of multicell spheroids in tissue culture as a model of nodular carcinomas. *J. Natl. Cancer Inst.* 46 113–120. 5101993

[B29] Sutlu T. Nyström S. Gilljam M. Stellan B. Applequist S. E. Alici E. (2012). Inhibition of intracellular antiviral defense mechanisms augments lentiviral transduction of human natural killer cells: implications for gene therapy. *Hum. Gene Ther.* 23 1090–1100. 10.1089/hum.2012.080 22779406 PMC3472531

[B30] Tillberg P. W. Chen F. Piatkevich K. D. Zhao Y. Yu C.-C. J. English B. P. (2016). Protein-retention expansion microscopy of cells and tissues labeled using standard fluorescent proteins and antibodies. *Nat. Biotechnol.* 34 987–992. 10.1038/nbt.3625 27376584 PMC5068827

[B31] Unnersjö-Jess D. Scott L. Blom H. Brismar H. (2016). Super-resolution stimulated emission depletion imaging of slit diaphragm proteins in optically cleared kidney tissue. *Kidney Int.* 89 243–247. 10.1038/ki.2015.308 26444032

[B32] Weigert M. Schmidt U. Boothe T. Müller A. Dibrov A. Jain A. (2018). Content-aware image restoration: pushing the limits of fluorescence microscopy. *Nat. Methods.* 15 1090–1097. 10.1038/s41592-018-0216-7 30478326

[B33] Zhong X. Gutierrez C. Xue T. Hampton C. Vergara M. N. Cao L.-H. (2014). Generation of three-dimensional retinal tissue with functional photoreceptors from human iPSCs. *Nat. Commun.* 5 1–14. 10.1038/ncomms5047 PMC4370190 24915161

