# Supplementary material for: High-Resolution Imaging of Tumor Spheroids and Organoids Enabled by Expansion Microscopy
Source: Front Mol Biosci. 2020 Sep 24;7:208. doi: 10.3389/fmolb.2020.00208 (PMC7543521; doi:10.3389/fmolb.2020.00208)
Supplement: Supplementary file 1 [file Table_1.DOCX]

| Figure | Sample Description | Immersion Cleared? | Expanded? | Post-fixation Staining | Denaturation method | Post-expansion Staining 1 | Post Expansion Staining 2 | Detection objective | Voxel Size (x,y,z μm) | Depth of section from surface (μm) |
| --- | --- | --- | --- | --- | --- | --- | --- | --- | --- | --- |
| 1b and b' | A498 cell spheroid transfected with mitoGFP bacmam | No | Yes | No | Proteinase-K | WGA (1:50) in PBST overnight at 37℃ | DAPI (1:1000) in PBST | W Plan-Apochromat 10x/0.5 | 0.644 x 0.644 x 4.00 | 308 |
|  |  |  |  |  |  |  |  |  |  |  |
| 2a, top left | GBM 18 spheroid expressing cytosolic tdTomato | Yes | No | No | N/A | N/A | N/A | LSFM Clearing 20x/1.0 | 0.229 x 0.229 x 1.00 | 104 |
| 2a, top right | GBM 18 spheroid expressing cytosolic tdTomato | No | Yes | No | N/A |  |  | W Plan-Apochromat 10x/0.5 | 0.645 x 0.645 x 2.12 | 381 |
| 2a, bottom left | GBM 18 spheroid expressing cytosolic tdTomato | Yes | No | anti-tdTomato (1:50, sicgen in blocking solution) AND donkey anti-sheep Star635p (1:25 Aberrior in blocking solution) overnight at 37℃ | N/A | N/A | N/A | LSFM Clearing 20x/1.0 | 0.229 x 0.229 x 1.00 | 97 |
| 2a, bottom right | GBM 18 spheroid expressing cytosolic tdTomato | No | Yes | No | N/A | anti-tdTomato (1:50, sicgen in blocking solution) AND anti-sheep Star635p (1:25 Aberrior® in blocking solution) overnight at 37℃ | N/A | W Plan-Apochromat 10x/0.5 | 0.645 x 0.645 x 2.12 | 437 |
|  |  |  |  |  |  |  |  |  |  |  |
| 3, top | A498 cell spheroid | No | Yes | No | Heat Disruption | Anti-Mic60 (1:100, proteintech® in PBST) AND Anti-rabbit Star635p (1:100, Abberior® in PBST) overnight at 37℃ | DAPI (1:1000 in PBST) | W Plan-Apochromat 10x/0.5 | 0.456 x 0.456 x 2.41 | 214 |
| 3, upper middle | A498 cell spheroid | No | Yes | No | Heat Disruption | Anti-p-histone (1:100, Cell Signalling Technology in PBST) AND (Anti-rabbit Star635p (1:100, Abberior®in PBST) overnight at 37℃ | DAPI (1:1000 in PBST) | W Plan-Apochromat 10x/0.5 | 0.457 x 0.457 x 2.54 | 203 |
| 3, lower middle | A498 cell spheroid | No | Yes | No | Heat Disruption | Anti-TUBA4A (1:100, Sigma-Aldrich in PBST) AND (Anti-goat Star635p (1:100, Abberior® in PBST) overnight at 37℃ | DAPI (1:1000 in PBST) | W Plan-Apochromat 10x/0.5 | 0.457 x 0.457 x 1.46 | 197 |
| 3, bottom | A498 cell spheroid | No | Yes | No | Heat Disruption | Anti-ARFGAP1 (1:100, ATLAS ANTIBODIES in PBST) AND (Anti-rabbit Star635p (1:100, Abberior® in PBST) overnight at 37℃ | DAPI (1:1000 in PBST) | W Plan-Apochromat 10x/0.5 | 0.457 x 0.457 x 2.30 | 196 |
|  |  |  |  |  |  |  |  |  |  |  |
| 4a | A498 cell spheroid | Yes | No | DAPI (1:10000 in blocking solution) | N/A | N/A | N/A | LSFM Clearing 20x/1.0 | 0.191 x 0.191 x 1.19 | 126 |
| 4b | A498 cell spheroid | No | Yes | No | Heat disruption | DAPI (1:1000 in PBST) | N/A | W Plan-Apochromat 10x/0.5 | 0.458 x 0.458 x 2.41 | 224 |
|  |  |  |  |  |  |  |  |  |  |  |
| 5a | MDCK II cyst | No | Yes | Anti-TUBA4A (1:100, Sigma-Aldrich in PBST) AND DAPI (1:1000 in PBST) overnight at 37℃ | Proteinase-K | N/A | N/A | W Plan-Apochromat 20x/1.0 | 0.229 x 0.229 x 1.00 | 119 |
| 5b | Retinoid electroporated with membrane GFP | No | Yes | anti-Otx2 (P-15) Polyclonal (Santa Cruz sc-30659 in blocking solution 1:50) AND anti-goat Star635p (1:50 Aberrior in blocking solution) overnight at 37℃ | Proteinase-K | N/A | N/A | W Plan-Apochromat 10x/0.5 | 0.645 x 0.645 x 2.56 | N/A |
| 5c | A498 cell spheroid expressing tdTomato and NK-92 GFP co-culture | No | Yes | No | Proteinase-K | anti-GFP (1:200, Invitrogen in blocking solution) AND (Anti-goat Star635p (1:100, Abberior® in blocking solution) overnight at 37℃ | N/A | W Plan-Apochromat 20x/1.0 | 0.229 x 0.229 x 1.00 | N/A |
