# Supplementary figures and images for: High-Resolution Imaging of Tumor Spheroids and Organoids Enabled by Expansion Microscopy
Source: Front Mol Biosci. 2020 Sep 24;7:208. doi: 10.3389/fmolb.2020.00208 (PMC7543521; doi:10.3389/fmolb.2020.00208)

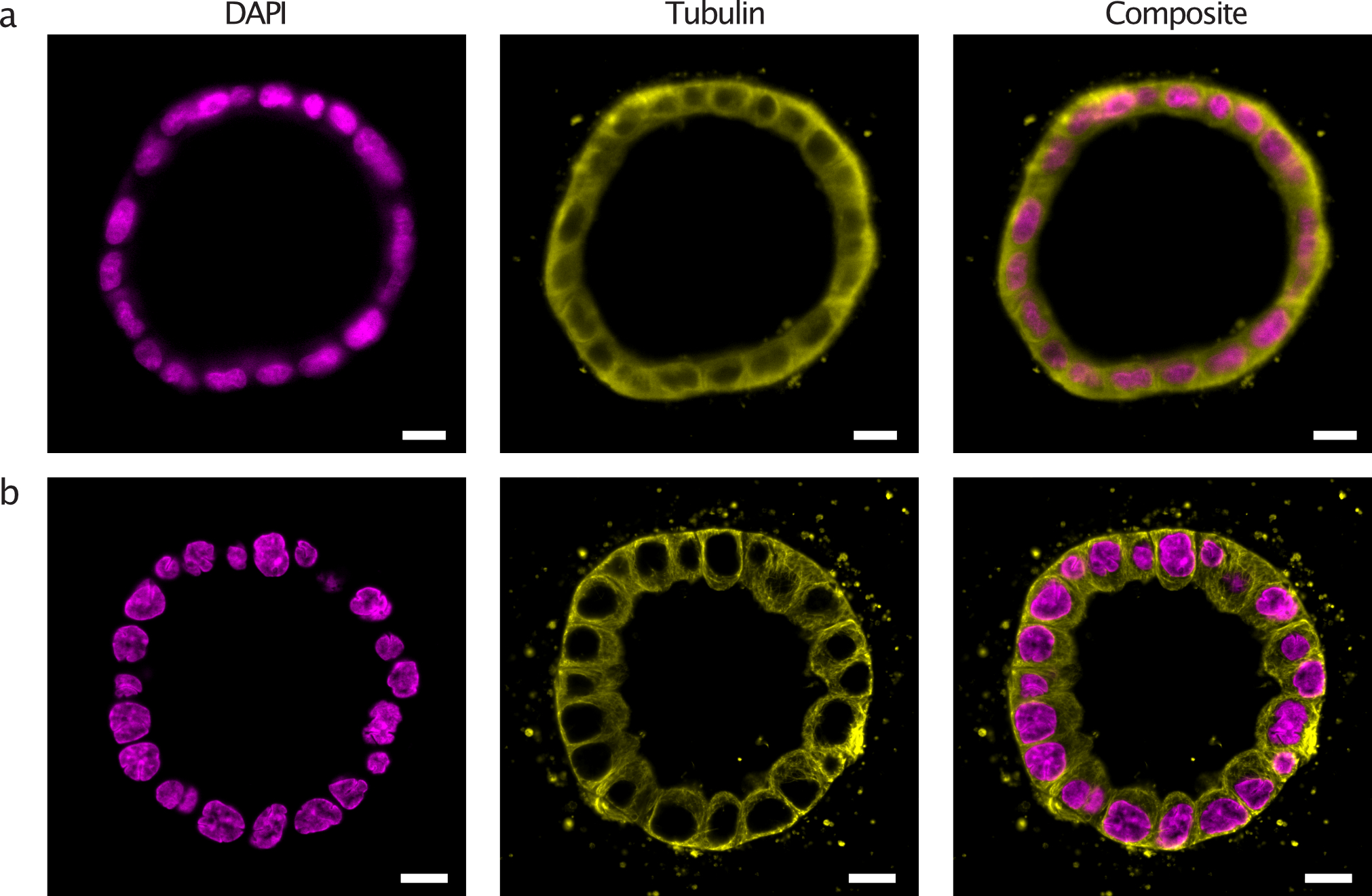

Supplement: Supplementary file 7 [file Image_1.jpg]
